# Analysis of ventricular repolarization parameters and heart rate variability in obesity: a comparative study

**DOI:** 10.1038/s41598-024-76580-x

**Published:** 2024-10-28

**Authors:** Akash Tomar, Himani Ahluwalia, H. S. Isser, Sameer Gulati, Puneet Kumar, Indrajeet Yadav

**Affiliations:** 1 Department of Physiology, Kasturba Medical College, Manipal Academy of Higher Education, Manipal, Karnataka 576 104 India; 2 Department of Physiology, Vardhman Mahavir Medical College and Safdarjung hospital, New Delhi, Delhi India; 3 Department of Cardiology, Vardhman Mahavir Medical College and Safdarjung hospital, New Delhi, Delhi India; 4 Department of Internal Medicine, Vardhman Mahavir Medical College and Safdarjung hospital, New Delhi, Delhi India; 5 Department of Physiology, Lady Hardinge Medical college, New Delhi, Delhi India; 6 Department of Physiology, Govt. Medical College, Ratlam, Madhya Pradesh India; 7 Department of Physiology, Kasturba Medical College, Manipal Academy of Higher Education, Manipal, Karnataka 576 104 India

**Keywords:** Obesity, Ventricular repolarization, Cardiac autonomic neuropathy, Heart rate variability, Electrocardiography, Cardiac remodeling, Cardiovascular biology, Cardiology

## Abstract

Obesity is associated with dysfunctional electrocardiographic and cardiac autonomic parameters, which may lead to increased cardiovascular morbidity. Novel electrocardiographic repolarization markers such as Tpeak-Tend (Tpe) interval have not yet been deeply studied in obese patients. We aimed to investigate the association between ventricular repolarization parameters and heart rate variability (HRV) and how they are affected by changes occurring in the cardiac autonomic nervous system. Ninety subjects categorized by Southeast Asian BMI (kg/m^2^) standards - normal (18-22.9), overweight (23-24.9), and obese (> 24.9), underwent assessment of ventricular repolarization parameters and HRV. Linear correlation between different parameters was also conducted. Obese subjects exhibited longer QTc and Tpe intervals compared to normal-weight subjects (p-value < 0.001, 0.026 respectively). The QTc interval showed a significant correlation (*p* < 0.05) with all HRV parameters by linear correlation, while the Tpe interval did not. Anthropometric parameters (BMI, WC, and WHR) were also correlated to both ventricular repolarization variables and HRV. While changes in the QTc interval may be due to obesity and/or autonomic changes occurring in the obese state, the Tpe interval does not show a relation with autonomic parameters. Thus, implicating that a change in the Tpe interval is primarily due to the direct effect of either cardiac or visceral obesity. The observed associations between ECG parameters, obesity indices, and HRV parameters suggest a role for ECG in screening for cardiovascular morbidity.

## Introduction

**Fig. 1:**
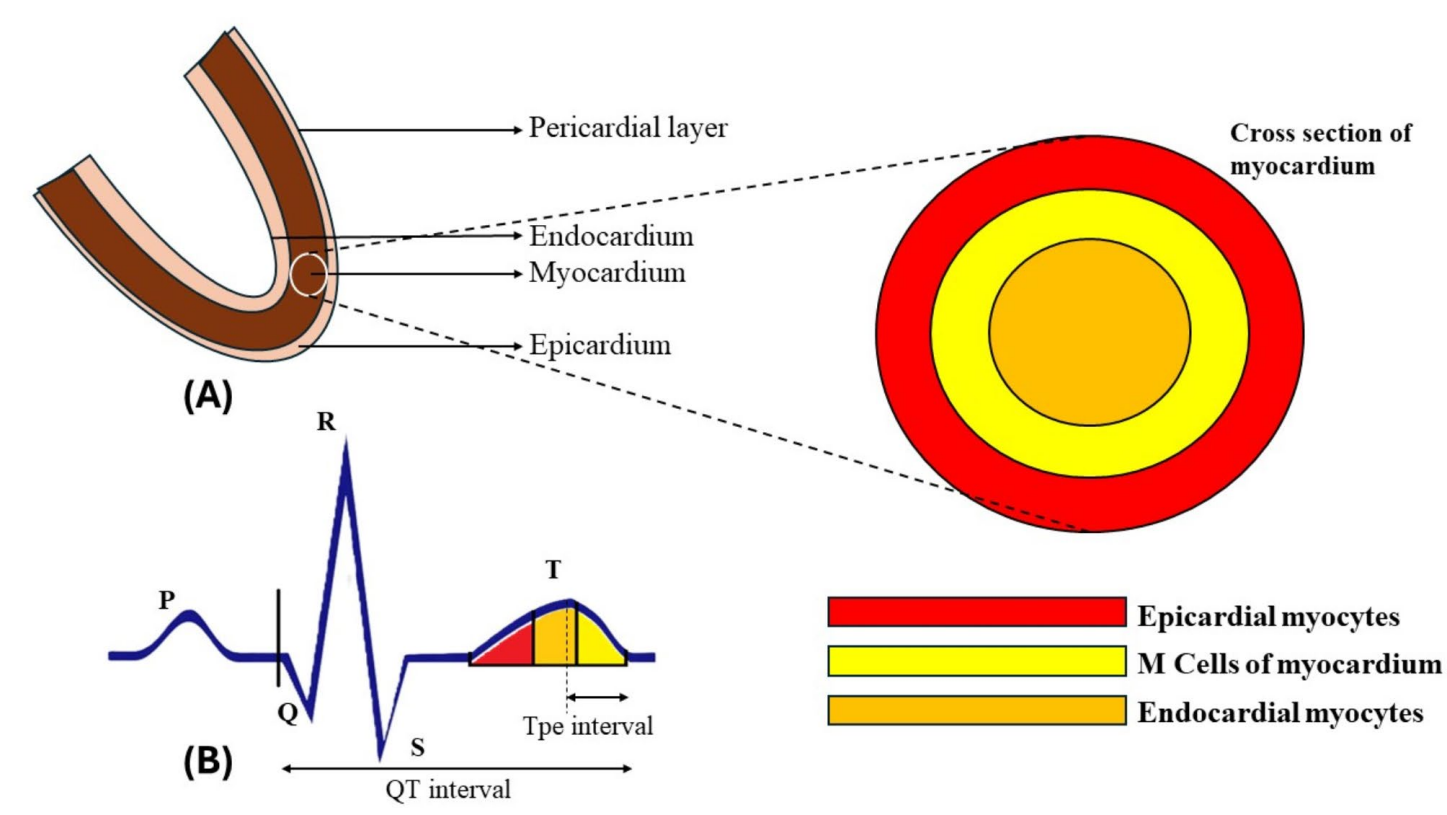
Section of myocardium showing different layers of the cardiac myocardium (**A**), ECG characteristics measured in our study, QT interval and Tpe interval (**B**), Effect of different layers of myocardium on Tpe interval (**B**).

Obesity is a well-established major risk factor for cardiovascular morbidity and is often linked to various cardiovascular events^1^. In Southeast Asian regions, obesity is emerging as a direct risk factor for cardiovascular and other systemic morbidities^1^. Obesity causes alterations in the structure of cardiac tissue, such as ventricular hypertrophy, left atrial enlargement, and cardiac remodeling at the molecular/cellular and interstitial level^2,3^. These changes affect the electrical events of depolarization and repolarization during the cardiac cycle, which are then reflected as changes in surface ECG, including prolonged duration of various segments (PR interval and QT interval) and decreased amplitude of the QRS complex. Recently, newer parameters like the Tpeak-to-Tend (Tpe) interval and JT interval are being recognized as better markers for ventricular repolarization^4,5^. This is because the Tpe interval, the time between the peak and the end of the T wave, has been established as a measure of transmural dispersion of ventricular repolarization^6^. The prolongation of the Tpe interval may be attributed to the structural effects of obesity on the heart, which profoundly impacts M cells in the myocardium (the mid-myocardial layer of the heart). A prolonged Tpe interval also implies that the myocardium spends more time in repolarization, making it more susceptible to arrhythmogenic stimuli since it is during this phase that the heart is in a state of relative refractory period (Fig. 1).

Obesity is linked to widespread enlargement of fat depots, systemic inflammation, and uncontrolled release of fatty acids (FAs) into the bloodstream^7^. These characteristics contribute to the development of cardiac adiposity, characterized by an increase in intramyocardial triglyceride content and the expansion of fat volume surrounding the heart and major blood vessels. Moreover, the metabolically active adipose tissue secretes pro-inflammatory and arrhythmogenic adipocytokines, which modulate ionic channel activity and prolong the action potential duration (APD) of the ventricles^8^.

Abnormalities in ventricular repolarization have been correlated with susceptibility to sudden cardiac death in patients who have recovered from myocardial infarction, patients with QT interval prolongation due to specific drugs, users of liquid protein-modified diets, individuals with idiopathic mitral valve prolapse, and patients afflicted with congenital long-QT syndrome^9^. These factors also pose risks for various arrhythmogenic states, such as long QT syndrome and Brugada syndrome^9^. Researchers have also demonstrated that the prolongation of ventricular repolarization is a concerning factor in the origin of life-threatening ventricular arrhythmias in both acquired and congenital conditions. An increased risk of arrhythmia leading to ventricular dysfunction is characterized by a prolonged QT interval, abnormal T-wave configuration, and increased dispersion of the QT interval, generally termed “long QT syndromes” (LQTS)^10^. It has also been shown that analyzing different components of the QT interval is more useful for recognizing the risk of sudden cardiac death than measuring the QT interval alone in electrocardiographic parameters^11^.

The autonomic nervous system (ANS) plays a critical role in maintaining cardiovascular homeostasis^12^. Autonomic nerve discharges (as in physiological conditions) lead to the local release of neurotransmitters that shorten the refractory period, thereby increasing repolarization^13^. Autonomic changes have a direct effect on the ventricular myocardium and therefore, impact the duration of cardiac repolarization^14^. The Framingham Offspring cohort revealed that individuals with abnormal heart rate variability (HRV), an indicator of sympathetic and parasympathetic activity in the heart, have a greater risk of developing clinical features of metabolic syndrome^12^. These individuals also faced a heightened incidence of diabetes, cardiovascular events, and mortality risk over a 20-year period. Obesity increases sympathetic activity while reducing parasympathetic (vagal) tone, indicating poor autonomic control of cardiac rhythm in obese individuals. The waist-to-hip ratio (WHR) serves as an indicator of visceral adiposity and is strongly correlated with increased sympathetic activity and reduced parasympathetic activity^15^. Pathology affecting the sympathetic and parasympathetic nerve fibers supplying the blood vessels and heart can lead to autonomic dysfunction^16^. As neuropathy tends to occur earlier in the longest nerve fibers, the first manifestations of autonomic neuropathy in metabolic disorders stem from parasympathetic nervous system (PNS) denervation and alterations in heart rate variability (HRV)^17^. These abnormalities in heart rate control and vascular tone typically manifest as resting tachycardia in the advanced stages of autonomic dysfunction.

With further advancement of this autonomic dysfunction, the signs of abnormal heart rate control and vascular tone become more prominent, manifesting as resting tachycardia. This progression eventually disrupts normal blood pressure regulation, resulting in presyncope symptoms, exercise intolerance, and palpitations. These symptoms not only significantly impair quality of life but also reflect cardiovascular instability in these groups of people^18^.

In recent times the development of cardiovascular symptoms and sudden cardiac events have been increasing in the younger population at an alarming rate^19^. This is a cause of concern and necessitates further research for a better understanding to tackle the emerging epidemic of cardiovascular morbidity in this age group. Thus, this study proposes to investigate the interplay between altered autonomic functions and cardiac ventricular repolarization parameters in populations of normal, overweight, and obese individuals. This could also provide insight into how obesity impacts cardiovascular parameters early in an obese person’s life.

## Methods

Our study was a cross-sectional study and sample size was calculated by a previous study done by Hussain et al.^20^.

Formula used –

$${\text{N}} = 2\:{\left\{\frac{\left(\frac{{\upsigma\:}1\text{Z}\text{a}}{2}\left(2\text{T}\right)+\:\text{Z}1-{\upbeta\:}\right)}{\left({\upmu\:}\text{A}\:-\:{\upmu\:}\text{B}\right)}\right\}}^{2}$$

T = number of pairwise comparison, if we have k groups then, k(k-1)/2 possible comparison: µ_A_ = 374.83 µ_B_ = 390.17 pooled σ = 18.4 T = 3, *n* = 31 so, per group = 16.

We took *n* = 30 in each group (BMI (kg/m^2^)- 18-22.9, 23-24.9 and > 24.9) to increase the power of our study further to 90%. This was in accordance with Mishra et al.^21^ who determined obesity cut-offs in the Southeast Asia region and suggested that BMI levels above 22.9 indicate a higher cardiovascular risk, different from Western populations where obesity is defined as a BMI of > 25.

The inclusion criteria for the study group were individuals within the range of 19–35 years of age. Only people with morning shifts of not more than 8 h, were considered for participation^22^. We excluded individuals who were on medications affecting ECG parameters, such as antimalarials (chloroquine, mefloquine), antipsychotics, ketoconazole, and antiarrhythmics. Subjects with chronic kidney disease, liver disease, endocrine abnormalities, cardiac abnormalities, severe anemia, substance abuse, hypertension, diabetes mellitus were also excluded from the study.

### Study protocol

Prior informed consent was obtained after explaining the study procedure to the subjects. Participants were advised to avoid caffeine or tea on the investigation day and to have a light breakfast at least three hours before testing. Subjects were referred to the Exercise Physiology Lab, Department of Physiology, Vardhman Mahavir Medical College. Investigations were conducted between 9 a.m. and 11:30 a.m. to account for variations in autonomic tone throughout the day^23^. The lab temperature was maintained between 23 °C and 25 °C. Participants were allowed to acclimatize to lab conditions for 20 min before testing began. Tests were performed uniformly across all participants to minimize bias.

### Parameters measured

Anthropometric measurements included height (using a stadiometer, in cm), weight (using an automatic weighing scale (Crown)™ in kg), and body mass index (BMI) calculated as weight (in kg) / height (in meters) ². Additional measurements included waist circumference (WC) at the umbilical level, hip circumference (HC) at the pubic symphysis and the largest outward part of the hip, and waist-hip ratio (WHR) calculated as WC/HC. ECG intervals namely Tpe, QT and JT intervals were recorded automatically using the ML 870B80 POWERLAB (8/30) (A.D. Instruments) in lead V5^24^. Tpe/QT, Tpe/QTc and Tpec/QTc ratios were also calculated manually, in lead v5 which is the lead preferred for measuring the Tpe interval as recommended by Emori et al.^25^. The correction of QT interval for heart rate was done automatically by the machine and the formula used was Bazette’s formula that is QTc = QT /√RR.

Heart rate variability (HRV) parameters were measured and analyzed using the built-in HRV module of ML 870B80 (A.D. Instruments). A 5-minute ECG recording was used to calculate time-domain parameters such as mean heart rate, SDRR (square root of the variance of the RR interval), SDSD (standard deviation of square root deviations of RR intervals), RMSSD (root mean square of standard deviation), MedRR (median of all RR intervals), and pRR50 (percentage of successive beats with a difference of more than 50 ms). Frequency domain parameters like total power (TP), very low frequency (VLF), low frequency (LF), high frequency (HF), and the LF/HF ratio were expressed as power (in ms²), normalized units (nu), and percentages (%).

### Statistical analysis

Data was analyzed using GraphPad Prism version 9.3.0 (GraphPad Software, San Diego, California, USA). The Kolmogorov-Smirnov (K-S) test was used to assess the normality of data distribution. Continuous variables were presented as Mean ± Standard deviation (SD). ANOVA was performed to assess differences among the normal weight, overweight, and obese groups. Post-hoc Tukey’s test was applied to determine statistically significant differences between groups. Pearson’s linear correlation was used to analyze relationships among anthropometric, ventricular repolarization, and HRV parameters. Significance was set at *p* < 0.05 with a 95% confidence interval and β = 0.2, providing a power of 80%.

### Ethical policy and institutional ethics committee

The study protocol was approved by the Institutional Ethics Committee (IEC/VMMC/SJH/Thesis/2019-10/CC-256) on August 17, 2021. The procedures adhered to the guidelines outlined in the Declaration of Helsinki 2013. Informed consent was obtained from all participants before enrollment.

## Results

Ninety subjects aged 19–35 years, were assessed according to the inclusion and exclusion criteria.

### Anthropometric parameters

Measured anthropometric parameters included weight, height, BMI, WC, HC, and WHR. Most of the anthropometric parameters (except Height) were statistically different between the three groups with higher values in the obese group when compared with the other two groups (Table 1).

**Table 1:** Baseline characteristics in the study groups.

	Normal (*n* = 30)	Overweight (*n* = 30)	Obese (*n* = 30)	*p* value
Age (years)	26.80 ± 5.25	27.33 ± 4.22	29.37 ± 3.75	0.068
Weight (kg)	57.53 ± 8.21	68.43 ± 7.34	81.93 ± 17.17	< 0.001^*^
Height (meters)	1.67 ± 0.09	1.69 ± 0.09	1.67 ± 0.11	0.817
BMI (kg/m^2^)	20.59 ± 1.27	23.89 ± 0.63	29.02 ± 3.44	< 0.001^*^
Waist circumference (cm)	81.27 ± 4.99	86.13 ± 5.30	99.67 ± 13.21	< 0.001^*^
Hip circumference (cm)	94.30 ± 4.72	98.03 ± 3.19	104.37 ± 8.53	< 0.001^*^
WHR	0.86 ± 0.03	0.88 ± 0.04	0.95 ± 0.05	< 0.001^*^

* p−value at significant level that is <0.05, Values mentioned as (Mean ± SD), ANOVA test for continuous variables. Abbreviations: WHR = Waist to Hip ratio, SD = Standard Deviation.

### Electrocardiographic and heart rate variability parameters

Electrocardiographic measurements showed higher heart rates in the obese and overweight groups when compared to the normal weight group (Table 2). The Tpe interval was also significantly different in the three groups with higher values in the obese group. The QTc interval also varied notably across the three study groups (Table 2).

Statistically significant differences were also observed in time-domain and frequency-domain parameters, including the average RR interval, median RR interval, LF power%, and VLF power% in the three groups (Table 2).

**Table 2:** Electrographic and Heart Rate Variability parameters in the study groups.

Parameters	Normal (*n* = 30)	Overweight (*n* = 30)	Obese (*n* = 30)	*p* value
Heart rate (bpm)	68.69 ± 10.57	74.34 ± 7.97	74.83 ± 8.59	0.018^*^
TPe interval (ms)	56.23 ± 3.81	57.18 ± 7.33	60.63 ± 7.58	0.026^*^
QT interval (ms)	341.08 ± 25.63	342.30 ± 17.23	345.86 ± 22.08	0.681
QTc interval (ms)	362.09 ± 17.36	380.17 ± 22.78	385.03 ± 25.01	< 0.001^*^
Tpe/QT ratio	0.17 ± 0.02	0.17 ± 0.02	0.18 ± 0.02	0.136
Tpe/QTc ratio	0.16 ± 0.01	0.15 ± 0.02	0.16 ± 0.02	0.429
Tpec/QTc ratio	0.159 + 0.002	0.161 + 0.004	0.164 + 0.003	0.657
JT interval (ms)	243.03 ± 25.48	244.53 ± 17.10	240.87 ± 42.76	0.724
AVERR	902.26 ± 133.78	837.94 ± 100.11	817.15 ± 89.92	0.009^*^
MEDRR	900.50 ± 134.71	837.01 ± 100.50	816.36 ± 90.94	0.011^*^
SDRR	49.64 ± 17.17	48.55 ± 17.85	44.88 ± 18.80	0.563
SDSD	49.28 ± 24.41	39.29 ± 20.17	38.48 ± 21.75	0.116
RMSSD	49.23 ± 24.38	39.41 ± 20.13	38.45 ± 21.73	0.12
PRR50	28.13 ± 23.11	20.82 ± 19.92	15.79 ± 17.04	0.064
TOTAL POWER	2634.30 ± 2002.35	2673.08 ± 1794.37	2146.24 ± 1895.99	0.491
VLF POWER	624.79 ± 486.73	862.18 ± 591.17	882.69 ± 692.79	0.183
LF POWER	760.77 ± 821.19	856.41 ± 931.42	490.44 ± 417.20	0.157
HF POWER	1201.25 ± 1031.22	931.94 ± 963.71	957.45 ± 1348.87	0.595
VLF POWER%	28.92 ± 14.96	37.07 ± 18.09	43.27 ± 17.09	0.006^*^
LF POWER%	27.62 ± 11.37	30.42 ± 11.70	23.01 ± 7.55	0.024^*^
HF POWER %	41.56 ± 17.83	31.81 ± 19.47	32.49 ± 16.97	0.072
LF POWER NU	41.34 ± 20.43	51.59 ± 19.32	43.94 ± 17.52	0.104
HF POWER NU	56.31 ± 18.60	47.49 ± 18.32	53.84 ± 16.77	0.151
LF/HF	1.16 ± 1.56	1.45 ± 1.08	1.21 ± 1.79	0.726

*p−value at significant level that is <0.05, Values mentioned as (Mean *±* SD), ANOVA test for continuous variables. Abbreviations: Tpe = T peak to T end, QTc = Corrected QT interval, VLF = Very low frequency, LF = Low frequency, HF = High frequency, LF(nu) = Low frequency in normalised units, HF(nu) = High frequency in normalised units, SD = Standard Deviation, AVRR = Average RR interval, MEDRR = median RR interval, SDRR = Standard deviation of RR intervals, SDSD = standard deviation of differences between adjacent RR intervals, RMSSD = square root of the mean squared differences of successive RR intervals, Prr50 = percentage of adjacent RR differing by more than 50ms.

### Pearson linear correlation of anthropometric and ventricular repolarization parameters with HRV parameters

Anthropometric parameters like BMI, WC, and WHR showed significant negative correlations with HF power in ms², nu, and %. Additionally, WHR and WC were correlated with time-domain HRV parameters (Table 3). While the QTc interval showed significant correlations with total power, HF power (ms²), HF power%, LF power (nu), HF power (nu), LF/HF ratio, average RR, MedRR, SDRR, SDSD, RMSSD, and pRR50, no correlation was observed between the Tpeak-Tend interval and any HRV parameters in this study (Table 3).

**Table 3:** Pearson linear correlation of anthropometric parameters and ventricular repolarization parameters with HRV parameters:

	BMI		WHR		WC		QTc		Tpe	
	*r*	*p*-value	*r*	*p*-value	*r*	*p*-value	*r*	*p*-value	*r*	*p*-value
Total Power	-0.341	0.076	-0.441	0.019^*^	-0.333	0.084	-0.374	0.0003^*^	-0.0507	0.6354
VLF Power (ms^2^)	-0.232	0.225	-0.287	0.131	-0.166	0.39	-0.0648	0.5443	0.143	0.1787
LF Power (ms2)	-0.255	0.183	-0.333	0.078	-0.251	0.189	-0.1561	0.1417	-0.1206	0.2575
HF Power (ms^2^)	-0.457	0.022^*^	-0.501	0.011^*^	-0.517	0.008^*^	-0.3722	0.0003^*^	-0.0257	0.8102
VLF Power %	0.326	0.079	0.408	0.025^*^	0.436	0.016^*^	0.3966	0.0001^*^	0.1408	0.1855
LF Power %	0.158	0.403	0.165	0.384	0.164	0.387	0.04731	0.6579	-0.1907	0.0718
HF Power %	-0.387	0.035^*^	-0.462	0.01^*^	-0.489	0.006^*^	-0.3755	0.0003^*^	-0.0257	0.81
LF Power(nu)	0.381	0.038^*^	0.468	0.009^*^	0.512	0.004^*^	0.2723	0.0094^*^	-0.0758	0.4775
Hf Power(nu)	-0.409	0.025^*^	-0.478	0.008^*^	-0.519	0.003^*^	-0.2635	0.0121^*^	0.05241	0.6237
LF/HF ratio	0.215	0.263	0.282	0.138	0.294	0.122	0.2292	0.0298^*^	0.00099	0.9927
AVRR	-0.503	0.005^*^	-0.657	< 0.001^*^	-0.646	< 0.001^*^	-0.5066	< 0.0001^*^	0.1164	0.2747
MEDRR	-0.5	0.005^*^	-0.652	< 0.001^*^	-0.644	< 0.001^*^	-0.5087	< 0.0001^*^	0.1153	0.2791
SDRR	-0.332	0.078	-0.397	0.033^*^	-0.354	0.06	-0.3841	0.0002^*^	0.07747	0.468
SDSD	-0.355	0.054	-0.437	0.016^*^	-0.432	0.017^*^	-0.449	< 0.0001^*^	0.07311	0.4935
RMSSD	-0.355	0.054	-0.437	0.016^*^	-0.864	0.017^*^	-0.4499	< 0.0001^*^	0.07111	0.5054
PRR50	-0.316	0.108	-0.438	0.022^*^	-0.415	0.031^*^	-0.4685	< 0.0001^*^	0.02486	0.8161

* p−value at significant level that is <0.05, Values mentioned as (Mean ± SD), ANOVA test for continuous variables. Abbreviations: r = correlation coefficient, BMI = Body mass index, WHR = Wasit to hip ratio, WC = Waist circumference, Tpe = T peak to T end, VLF = Very low frequency, LF = Low frequency, HF = High frequency, LF(nu) = Low frequency in normalised units, HF(nu) = High frequency in normalised units, SD = Standard Deviation, AVRR = Average RR interval, MEDRR = median RR interval, SDRR = Standard deviation of RR intervals, SDSD = standard deviation of differences between adjacent RR intervals, RMSSD = square root of the mean squared differences of successive RR intervals, Prr50 = percentage of adjacent RR differing by more than 50ms.

## Discussion

### Relationship between ventricular repolarization parameters and obesity

Prolonged QT and Tpe intervals in obese individuals suggest altered cardiac electrophysiology, as indicated by increased durations of both ventricular depolarization and repolarization. However, since the Tpe/QT ratio is also higher in obese people, this may imply that the repolarization duration of the M cells of the myocardium contributes more to the total duration of the QT interval. A recent study done by Dykeirt et al.^26^ has shown that an increase in Tpe interval and its dispersion was longer in the obese group. Yamaguchi et al.^5^ documented that longer Tpe interval values are directly related to increased chances of cardiac morbidity (arrhythmia). Additionally, a study by Panikkath et al.^27^ stated that the risk of cardiac mortality (sudden cardiac death) increases with longer Tpe interval durations. Our study also corroborates this finding of longer Tpe values in obese individuals. There is not a clear consensus about normal Tpe values in different populations, however a relative increase in this interval has been associated with many cardiovascular comorbidities such as coronary heart disease, cerebrovascular disease, rheumatic heart disease^28^. The QTc interval was significantly longer in the obese on the electrocardiogram. Other ventricular repolarization parameters, such as the QT interval, Tpe/QTc ratio, Tpe/QT ratio, and JT interval, had longer durations in the obese but were not statistically significant between the three groups. A significant positive correlation was found between obesity indices (such as BMI, WC, and WHR) and ventricular repolarization parameters (such as the TPE interval and QTc interval) when all 90 subjects were analyzed together using Pearson linear correlation as shown in Table 3.

### Relationship between heart rate variability and obesity

In our study, the average RR interval [normal weight group (902.26 ± 133.78 ms), overweight group (837.94 ± 100.11 ms), and obese group (817.15 ± 89.92 ms)], was shorter in obese subjects compared to normal-weight subjects and overweight subjects. We found similar results with MedRR. The simple linear correlation of the average RR and MedRR intervals was also statistically significantly negatively correlated with BMI, WC, and WHR, further reinforcing that heart rate increases with body weight.

In our study, SDSD was significantly negatively correlated with both WC (*r* = -0.432, p-value = 0.017) and WHR (*r* = -0.437, p-value = 0.016). This has also been documented by Poliakova et al.^29^, who studied 97 subjects with no endocrinological or cardiovascular abnormalities and related obesity indices to heart rate variability. We also found a negative correlation between SDRR and WHR (*r* = -0.397, p-value = 0.033). SDSD and SDRR represent both SNS and PNS activity in humans and are highly correlated with VLF band power, LF band power, and total power in the measurement of short-term heart rate variability^30^. Thus, a negative correlation of these parameters with obesity indices indicates decreased activity of the autonomic nervous system. A previous study by Kleiger et al.^31^ indicates that higher SDRR values reduce the risk of mortality in acute myocardial infarction patients (5.3 times) compared to those with lower SDRR. We also documented a negative correlation between WHR and RMSSD (*r* = -0.437, p-value = 0.016), which has been previously documented by Kim et al.^32^, who found a strong negative correlation between WHR and RMSSD. Additionally, in our study, RMSSD was negatively associated with WC (*r* = -0.864, p-value = 0.017), consistent with findings from previous studies by Windham et al.^33^. Since RMSSD is an index for parasympathetic function or vagal influence on the heart, lower RMSSD values indicate decreased PNS activity in the obese. The pRR50 is an index for measuring high-frequency parameters in the heart and is closely correlated with PNS activity. This is considered a more reliable index than short-term SDNN measurements for short samples of 5-minute duration^34^. We also found that WHR and WC were significantly negatively correlated with pRR50 (*r* = -0.438, p-value = 0.022; *r* = -0.415, p-value = 0.031, respectively). Similar observations have also been documented in a study by Farah et al.^35^.

The observations of reduced indices of parasympathetic activity, such as RMSSD and pRR50 in the time domain and HF in the frequency domain, imply that obesity significantly reduces parasympathetic activity compared to normal-weight and overweight individuals. A higher LF/HF ratio despite significantly lower LF and LF power% in obese people also suggests that there was a derangement of both sympathetic and parasympathetic limbs of the autonomic nervous system in obesity, with parasympathetic derangement being more pronounced than sympathetic derangement.

A previous study by Rajalakshmi et al.^36^ showed that HF power (ms²) was negatively correlated with BMI (*r* = -0.40), WC (*r* = -0.37), and WHR (*r* = 0.37), as well as the negative correlation of HF (nu) with BMI (*r* = 0.32) and WC (*r* = 0.28), like what we found in our study (Table 3). The HF band, also known as the respiratory band, correlates with the respiratory cycle and reflects parasympathetic activity. It has been observed that total vagal blockade effectively eliminates HF oscillations^37^. The modulation of vagal tone thus plays a vital role in maintaining dynamic autonomic regulation crucial for cardiovascular health. Subsequently, deficient vagal inhibition has been causally linked to increased cardiovascular morbidity^38^. Therefore, the findings observed in our study show that as obesity increases, parameters measured in the HF band, such as HF power, HF power%, and HF nu, decrease. This suggests that obese subjects have reduced parasympathetic activity and increased sympathetic activity compared to normal-weight and overweight subjects as seen in our study.

### Relationship between heart rate variability and ventricular repolarization parameters

**Fig. 2:**
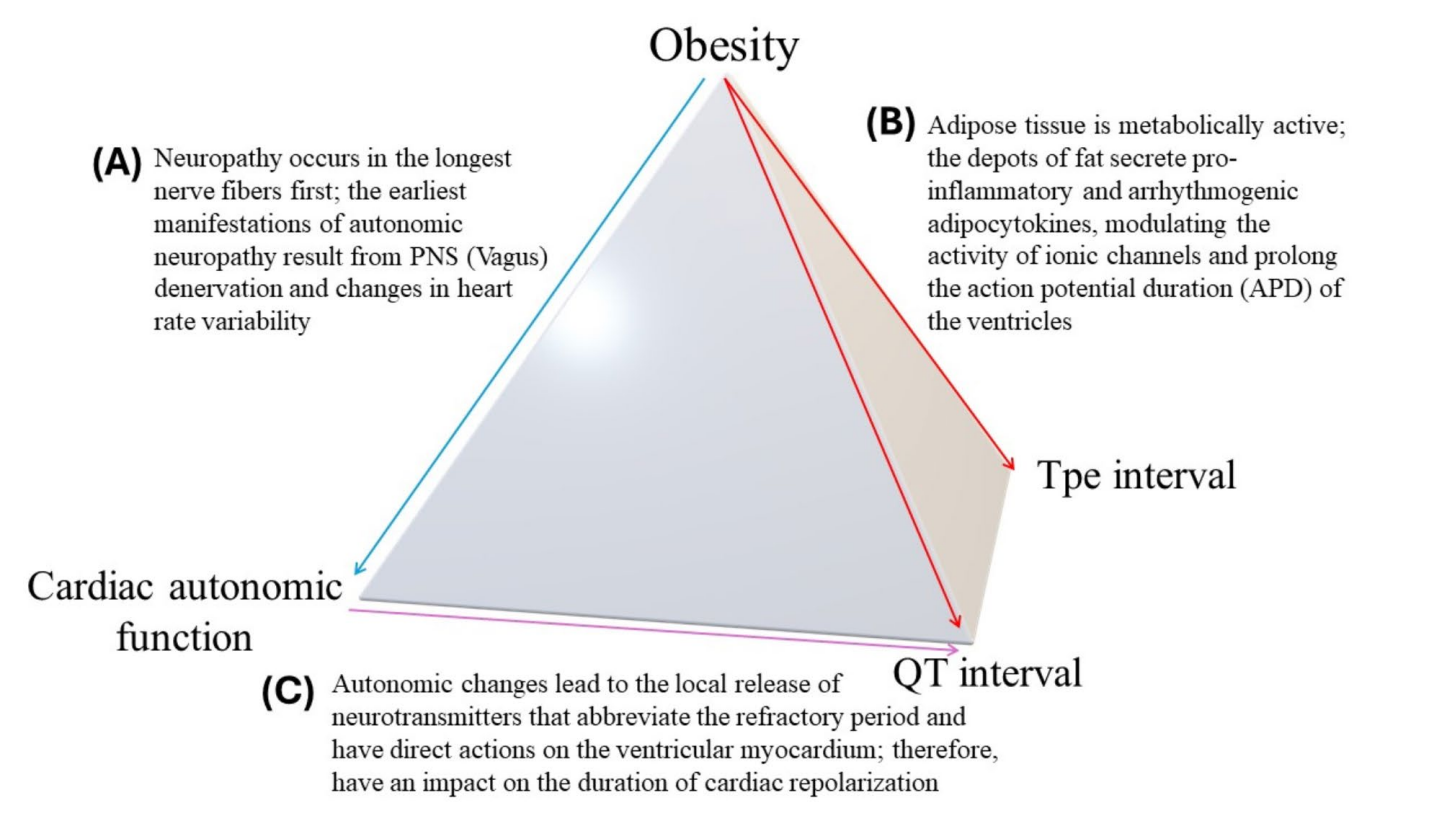
The effect of Obesity on Cardiac autonomic function (**A**), the effect of obesity on ventricular repolarisation parameters both QTc interval and Tpe interval (**B**), the effect of autonomic dysfunction on QT interval alone (**C**).

We also documented a statistically significant linear correlation between the QTc interval and various HRV parameters. A statistically significant negative correlation was found between the QTc interval and average RR, median RR, SDRR, SDSD, RMSSD, and pRR50 in time domain parameters. Additionally, a statistically significant negative correlation was found between the QTc interval and total power, HF power (ms²), HF power%, and HF nu in frequency domain parameters. A statistically significant positive correlation of the QTc interval was found with VLF power%, LF nu, and the LF/HF ratio. These findings suggest that the QTc interval is affected by autonomic activity in the heart, as shown by Maule et al.^39^ and Seon^40^. While no significant correlation between these HRV parameters and the TPE interval was observed, the potential mechanism explaining the association between a prolonged QTc interval and decreased HRV has not been fully established. Proposed mechanisms for QTc interval prolongation include gene mutations affecting myocardial ion channels involved in cardiac repolarization, an imbalance of the cardiovascular autonomic nervous system, hyperglycemia or hypoglycemia, hyperinsulinemia, obesity, post-myocardial infarction scar tissue, and ventricular hypertrophy^41–47^. A proposed mechanism of the effect of obesity on various parameters measured in our study is depicted in Fig. 2 which may be useful tool for further studies on the subject and can be validated in larger populations.

In developing countries screening the patients with ECG is an accessible and affordable tool for the masses. As we have seen that the Southeast Asian population is at greater risk for cardiovascular morbidities even at lower BMI levels. Therefore, using ventricular repolarization parameters is a cost-effective approach, especially in low- to middle-income countries, to assess whether a person might be at risk for cardiovascular disease, even in the absence of any genetic or predisposing conditions that could inherently cause an increase in ventricular repolarization parameters.

### Limitations

Future studies with larger cohorts may provide more insights into the causal relationship between QTc prolongation and HRV. This study did not report the prevalence of death or future cardiovascular disease associated with the studied variables. This cross-sectional study cannot determine cause and effect and could be influenced by other unmeasured factors. Echocardiography of the study population could provide more information on the structural changes in the heart and help better understand the relationship between the autonomic and structural effects of obesity. We chose to focus on Ventricular repolarization parameters as they are more accessible and affordable with decent reliability. Further studies using these specific tests could shed more light on these findings.

## Conclusion

The present study demonstrates a significant impact of obesity on the electrocardiographic and autonomic changes within the cardiovascular system. Such changes may result from hemodynamic, structural, and other alterations associated with obesity. The observed effects of obesity on the QT and QTc intervals appear to be linked to these changes, as evidenced by correlations with both anthropometric and heart rate variability (HRV) parameters. This suggests that a reduction in parasympathetic activity, coupled with relatively increased sympathetic activity, may contribute to QTc interval prolongation. Although the Tpe interval is less influenced by autonomic modulation, the data indicates that it serves as a crucial marker of altered electrophysiological function in M cells of the myocardium, directly impacted by obesity. These findings underscore the need for further studies to explore potential clinical applications.

## Data Availability

The datasets used and/or analysed during the current study available from the corresponding author on reasonable request.
